# Understanding the sodium cation conductivity of human epileptic brain tissue

**DOI:** 10.1063/5.0041906

**Published:** 2021-04-16

**Authors:** David Emin, Aria Fallah, Noriko Salamon, William Yong, Andrew Frew, Gary Mathern, Massoud Akhtari

**Affiliations:** 1 Department of Physics and Astronomy, University of New Mexico, Albuquerque, New Mexico 87131, USA; 2 Departments of Neurosurgery and Pediatrics, David Geffen School of Medicine, University of California at Los Angeles, Los Angeles, California 90095, USA; 3 Department of Radiology, David Geffen School of Medicine, University of California at Los Angeles, Los Angeles, California 90095, USA; 4 Department of Pathology, David Geffen School of Medicine, University of California at Los Angeles, Los Angeles, California 90095, USA; 5 Human Brain Mapping Center, David Geffen School of Medicine, University of California at Los Angeles, Los Angeles, California 90095, USA; 6 Semel Institute for Neuroscience and Human Behavior, David Geffen School of Medicine, University of California at Los Angeles, Los Angeles, California 90095, USA

## Abstract

Transient and frequency-dependent conductivity measurements on excised brain-tissue lesions from epilepsy patients indicate that sodium cations are the predominant charge carriers. The transient conductivity ultimately vanishes as ions encounter blockages. The initial and final values of the transient conductivity correspond to the high-frequency and low-frequency limits of the frequency-dependent conductivity, respectively. Carrier dynamics determines the conductivity between these limits. Typically, the conductivity rises monotonically with increasing frequency. By contrast, when pathology examinations found exceptionally disorganized excised tissue, the conductivity falls with increasing frequency as it approaches its high-frequency limit. To analyze these measurements, excised tissues are modeled as mixtures of “normal” tissue within which sodium cations can diffuse and “abnormal” tissue within which sodium cations are trapped. The decrease in the conductivity with increasing frequency indicates the predominance of trapping. The high-frequency conductivity decreases as the rate with which carriers are liberated from traps decreases. A relatively low conductivity results when most sodium cations remain trapped in “abnormal” brain tissue, while few move within “normal” brain tissue. Thus, the high densities of sodium nuclei observed by ^23^Na-MRI in epilepsy patients’ lesions are consistent with the low densities of diffusing sodium cations inferred from conductivity measurements of excised lesions.

## I. INTRODUCTION

Brain tissue of 67 epilepsy patients at the UCLA medical center was excised from a variety of lesion locations. Each freshly excised sample was subjected to (1) pathology examination, (2) measurement of the diffusion MRI [spin-echo nuclear magnetic resonance (NMR)] of its hydrogen nuclei, and (3) measurement of its frequency-dependent conductivity *σ*(*ω*). Qualitative changes in *σ*(*ω*) occur for excised samples whose pathology examination finds to be exceptionally disorganized. The following five paragraphs summarize the previously published experimental findings. The remainder of this paper develops a theoretical framework with which to understand these experimental results.

Transient currents induced by application of dc electric fields to cm-sized samples decayed in about 100 s as charge transport stopped at cellular blockages separated by about 0.5 mm.^1^ In addition, the conductivity measured between 6 and 1000 Hz typically rises gently with increasing frequency as reversals of the applied electric field increasingly enable drifting charges to avoid blockages. This charge transport was attributed to the slow motion of solvated ions, primarily solvated sodium cations.^1^ A density of solvated sodium cations of 2.5 × 10^25^ ions/m^3^ moving with a diffusion constant of 10^−9^ m^2^/s generates a room-temperature conductivity of about 0.16 S/m.^1^

Solvation occurs as oxygen atoms at the apex of the surrounding water molecules orient themselves toward cations.^2^ Thus, motion of solvated ions is associated with the reorientation of surrounding water molecules. Diffusion MRI (i.e., spin-echo NMR) performed on excised samples’ hydrogen atoms yields a diffusion constant of about 10^−9^ m^2^/s.^3^ The value of this diffusion constant is comparable to that inferred from conductivity measurements of solvated sodium cations. This result suggests that the diffusion constant obtained from these diffusion-MRI measurements monitors the reorientation of water molecules associated with the motion of solvated cations.^1^

In most instances, the conductivity rises as the applied frequency was increased, ∂*σ*(*ω*)/∂*ω* > 0. At high enough frequencies (typically >100 Hz), the frequency dependence of the conductivity weakens as most diffusing cations avoid blockages. The conductivity then simply becomes proportional to the product of the density of diffusing ions and their diffusion constant. Distinctively, the relative variations in the measured conductivities were an order of magnitude greater than those of diffusion constants obtained from diffusion-MRI measurements.^4^ Thus, changes in the ionic conductivity are overwhelmingly caused by changes in the density of diffusing ions.^4^

Although the conductivities of samples of excised brain tissue usually increase slowly with increasing frequency, the conductivities of some samples *decrease* with increasing frequency, ∂*σ*(*ω*)/∂*ω* < 0, at high frequencies (cf. Fig. 1 of Ref. 5). Furthermore, the magnitudes of conductivities at 100 Hz of these samples were somewhat lower than those of typical samples. As exemplified in Fig. 2 of Ref. 5, pathology investigations indicated that samples of brain tissue whose conductivities manifest this “anomalous” frequency dependence were especially disorganized.^5^ In particular, gray matter is extremely sparse and inhomogeneous, while white matter is grossly segregated.

Thus, measurements of the frequency-dependent conductivity imply that the densities of conducting sodium cations are especially low for especially disorganized brain tissue excised from epilepsy patients. By contrast, unusually, high concentrations of sodium atoms measured *in vivo* by ^23^Na-MRI are used to identify the lesions of epileptic patients.^6^ However, ^23^Na-MRI provides no information about the mobility of the sodium atoms it identifies.^7^ An aim of this paper is to address the relationship between the frequency-dependent sodium-cation conductivities *σ*(*ω*) measured on excised epileptic human brain tissue and sodium concentrations in epileptic lesions as measured by ^23^Na-MRI.

To analyze published measurements, we model excised samples as mixtures of “normal” tissue and “abnormal” (severely disorganized) tissue. Normal tissue generally exists at the margins of excised tissue and also may be included within its lesion. At its most extreme (*R*_*a*_ = 0 in the formulas of Sec. II), the model developed in Sec. II envisions sodium cations to be mobile within normal tissue and trapped within abnormal (severely disordered) tissue. The frequency-dependent ionic conductivity is then primarily governed by two electric-field-induced rates. First, *R*_*n*_ is the rate at which sodium cations in the normal material migrate to blockages at which they are stopped. Second, *R*_*l*_ is the rate at which ions are liberated from traps in the abnormal material to then move within the normal material. We find that the sign of the slope of the conductivity with respect to the applied frequency, *∂σ*(*ω*)/*∂ω*, in the high-frequency limit just depends on the ratio *R*_*l*_/*R*_*n*_ and on *f*, the fraction of ions initially trapped in the abnormal material. For inefficient transfer of ions from abnormal tissue into normal tissue, *R*_*l*_/*R*_*n*_ ≪ 1, the anomalous frequency-dependence of the high-frequency conductivity, ∂*σ*(*ω*)/∂*ω* < 0, only occurs when most ions are initially trapped in abnormal tissue, *f* → 1. Concomitantly, relatively few sodium cations initially move through normal tissue.

In summary, sodium-cation conductivities fall with increasing applied frequency to especially low values in the severely disorganized excised brain tissue excised from the most severely affected patients.^5^ The calculation in Sec. II implies that the density of sodium cations that then move in normal excised brain tissue has decreased. However, the total density of sodium nuclei measured by ^23^Na-MRI increases with epilepsy’s severity.^6^ In combination, these results imply that the density of *trapped* sodium cations rises with the severity of the structural disruptions associated with epilepsy. Discussion of the uncertainties and limitations of this simple model is relegated to Sec. III.

## II. CALCULATION

We divide brain tissue into two categories, “normal” (less etiologically related) and “abnormal” (more etiologically related), based on the severity of their structural disruptions. Most generally, ions can move within each category until being blocked by their respective cell structures. Thus, each category is treated as composed of polarization centers defined by their respective blockages. Ions’ motion within normal and abnormal polarization centers in addition to transfer from abnormal to normal tissue generates their frequency-dependent conductivities.

The application of an electric field at *t* = 0 initiates processes that alter the probabilities of cations (1) moving in the normal material (*P*_*n*_), (2) moving in the abnormal material (*P*_*a*_), and (3) being stopped and bound at blockages (*P*_*b*_). In particular, three master equations govern the temporal evolution of the occupation probabilities for cations moving in normal tissue, in abnormal tissue, and being stopped at cellular blockages,

$$\frac{dP_{n}\left(t\right)}{dt}=R_{l}P_{a}-R_{n}P_{n},$$ (1)

$$\frac{dP_{a}\left(t\right)}{dt}=-R_{l}P_{a}-R_{a}P_{a}=-\left(R_{l}+R_{a}\right)P_{a},$$ (2)

and

$$\frac{dP_{b}\left(t\right)}{dt}=R_{n}P_{n}+R_{a}P_{a}.$$ (3)

Here, *R*_*l*_ represents the rate characterizing the electric-field-induced liberation of cations from being trapped within abnormal structures. Liberated cations then transfer to normal matter within which their motion is relatively rapid. The rates with which cations move to blockages within normal and abnormal structures are denoted by *R*_*n*_ and *R*_*a*_, respectively.

The first-order linear differential equations [Eqs. (2) and (1)] have the respective solutions as follows:

$$P_{a}\left(t\right)=A\;\exp\left[-\left(R_{l}+R_{a}\right)t\right]$$ (4)

and

$$P_{n}\left(t\right)=B\;\exp\left(-R_{n}t\right)+A\left(\frac{R_{l}}{R_{n}-R_{l}-R_{a}}\right)\exp\left[-\left(R_{l}+R_{a}\right)t\right].$$ (5)

The coefficients *A* and *B* are determined from the initial conditions that *P*_*a*_ = *f* and *P*_*n*_ = 1 − *f* at *t* = 0, where *f* equals the fraction of cations initially occupying abnormal tissue and 1 − *f* equals the fraction of cations initially occupying normal tissue. Then, after some straightforward algebra, Eqs. (4) and (5) become

$$P_{a}\left(t\right)=f\;\exp\left[-\left(R_{l}+R_{a}\right)t\right]$$ (6)

and

$$P_{n}\left(t\right)=\frac{\left[R_{l}-\left(1-f\right)\left(R_{n}-R_{a}\right)\right]\exp\left(-R_{n}t\right)-fR_{l}\;\exp\left[-\left(R_{l}+R_{a}\right)t\right]}{R_{l}-R_{n}+R_{a}}.$$ (7)

Unlike Eq. (12) of Ref. 5, Eq. (7) includes the possibility of cation transport through the damaged regions. As such, Eq. (7) reduces to Eq. (12) of Ref. 5 in the limit that *R*_*a*_ = 0.

The transient ionic conductivity most generally is the sum of (1) that from mobile cations drifting through regions of normal tissue until they are stopped at blockages and (2) that from mobile cations drifting through damaged regions until being stopped at their blockages,

$$\sigma_{t}\left(t\right)=\sigma_{n}P_{n}\left(t\right)+\sigma_{a}P_{a}\left(t\right).$$ (8)

Here, *σ*_*n*_ and *σ*_*a*_ denote the initial ionic conductivities of normal tissue and abnormal tissue, respectively, where they are occupied by the totality of mobile ions, *n*_*c*_. Specifically, *σ*_*n*_ = *n*_*c*_(*q*^2^/*kT*)*D*_*n*_ and *σ*_*a*_ = *n*_*c*_(*q*^2^/*kT*)*D*_*a*_, where *q* represents the ion’s charge; *k* denotes the Boltzmann constant; *T* signifies the temperature; and *D*_*n*_ and *D*_*a*_ denote the ionic diffusion constants in normal and abnormal tissues, respectively. Employing Eqs. (6) and (7), the transient ionic conductivity [Eq. (8)] is written as

$$\begin{array}{ll} \sigma_{t}\left(t\right)= & \sigma_{n}\frac{\left[R_{l}-\left(1-f\right)\left(R_{n}-R_{a}\right)\right]\exp\left(-R_{n}t\right)-fR_{l}\;\exp\left[-\left(R_{l}+R_{a}\right)t\right]}{R_{l}-R_{n}+R_{a}}\\ & +\sigma_{a}f\;\exp\left[-\left(R_{l}+R_{a}\right)t\right]. \end{array}$$ (9)

Consider two limiting situations. First, if there are no damaged regions, *f* = 0, the transient conductivity, *σ*_*t*_(*t*) ∝ *σ*_*n*_ exp(−*R*_*n*_*t*), falls monotonically with time as solvated cations move through the normal material until they are stopped at a blockage. Second, if all the tissue is damaged with no transfer of trapped cations in abnormal tissue to normal tissue being possible, *f* = 1 and *R*_*l*_ = 0, the transient conductivity becomes simply *σ*_*t*_(*t*) ∝ *σ*_*a*_ exp(−*R*_*a*_*t*). That is, the transient current density falls monotonically with time as solvated cations move relatively slowly, *R*_*a*_ ≪ *R*_*n*_, through the damaged material until they are stopped at blockages.

In between these two extreme limits, the transfer of sodium cations from abnormal tissue to normal tissue can produce a qualitatively different behavior, trap-limited motion through normal tissue. For example, in the absence of transport in abnormal tissue, *σ*_*a*_ = 0 and *R*_*a*_ = 0, Eq. (9) becomes

$$\begin{array}{ll} \sigma_{t}\left(t\right) & =\sigma_{n}\frac{\left[R_{l}-\left(1-f\right)R_{n}\right]\exp\left(-R_{n}t\right)-fR_{l}\;\exp\left[-R_{l}t\right]}{R_{l}-R_{n}}\\ & =\sigma_{n}\left[\exp\left(-R_{n}t\right)+f\frac{R_{n}\exp\left(-R_{n}t\right)-R_{l}\exp\left[-R_{l}t\right]}{R_{l}-R_{n}}\right]. \end{array}$$ (10)

As indicated in Fig. 4 of Ref. 5, at short times, the transient current of Eq. (9) *rises* with time for sufficiently large *f* with *fR*_*l*_ > *R*_*n*_(1 − *f*),

$$\begin{array}{ll} & \sigma_{t}\left(t\right)\to \sigma_{n}\left\{\left(1-f\right)+t\left[-R_{n}+f\frac{\left(R_{l}-R_{n}\right)\left(R_{l}+R_{n}\right)}{\left(R_{l}-R_{n}\right)}\right]\right\}\\ & \;=\sigma_{n}\left\{\left(1-f\right)+t\left[fR_{l}-R_{n}\left(1-f\right)\right]\right\}, \end{array}$$ (11)

even though the transient current of Eq. (10) falls toward zero at sufficiently long times.

These qualitatively different time dependences of transient conductivity manifest themselves in qualitatively different frequency dependences of the frequency-dependent conductivity. Indeed, the frequency-dependent current density is obtained from transforming the transient current density as follows:

$$\sigma \left(\omega \right)\equiv \omega \int_{0}^{\infty }dt\sigma_{t}\left(t\right)\sin\left(\omega t\right).$$ (12)

Inserting Eq. (9) into this formula and performing the standard integrations yield

$$\begin{array}{ll} \sigma \left(\omega \right)= & \sigma_{n}\left\{\frac{\left[R_{l}-\left(1-f\right)\left(R_{n}-R_{a}\right)\right]}{R_{l}-R_{n}+R_{a}}\left(\frac{\omega^{2}}{{R_{n}}^{2}+\omega^{2}}\right)\right.\\ & \left.-f\left(\frac{R_{l}}{R_{l}-R_{n}+R_{a}}-\frac{\sigma_{a}}{\sigma_{n}}\right)\left[\frac{\omega^{2}}{{\left(R_{l}+R_{a}\right)}^{2}+\omega^{2}}\right]\right\}. \end{array}$$ (13)

Some limiting values of Eq. (13) can be readily discerned. In the low-frequency limit, *ω* → 0, the ac conductivity approaches the final value, *t* = ∞, of the transient conductivity, where all carriers are stopped by blockages: *σ*(0) = 0. In the high-frequency limit, *ω* → ∞, the ac conductivity approaches the initial, *t* = 0, value of the transient conductivity,

$$\sigma \left(\infty \right)=\sigma_{n}\left(1-f\right)+\sigma_{a}f.$$ (14)

In the absence of ionic transfer from abnormal tissue to normal tissue, *R*_*l*_ → 0, the frequency-dependent conductivity becomes simply the sum of that from polarization centers in the two types of tissues as follows:

$$\sigma \left(\omega \right)=\sigma_{n}\left(1-f\right)\left(\frac{\omega^{2}}{{R_{n}}^{2}+\omega^{2}}\right)+\sigma_{a}f\left[\frac{\omega^{2}}{{R_{a}}^{2}+\omega^{2}}\right].$$ (15)

Since ionic transport in extremely damaged excised brain tissue is relatively poor, *σ*_*a*_ ≪ *σ*_*n*_, the ionic conductivity of Eq. (15) becomes extremely small as the fraction of cations in damaged tissue grows, *f* → 1. By contrast, as *R*_*l*_ → ∞, all trapped carriers are readily liberated from abnormal tissue and are thereafter free to move through normal tissue. The effects of trapping then disappear from Eq. (13) as it reverts to that for all carriers moving through normal tissue,

$$\sigma \left(\omega \right)=\sigma_{n}\left(\frac{\omega^{2}}{{R_{n}}^{2}+\omega^{2}}\right).$$ (16)

**FIG. 1.**
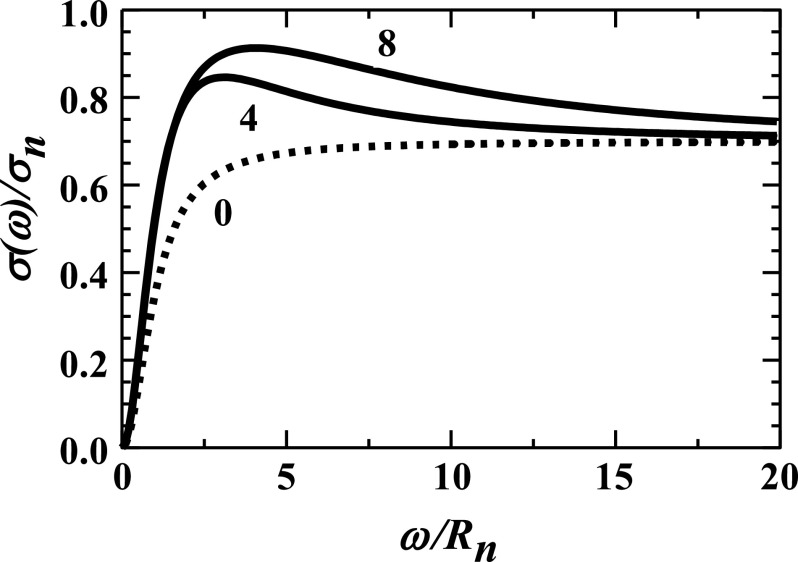
The relative ionic conductivities, *σ*(*ω*)/*σ*_*n*_, from Eq. (13) are plotted vs relative frequency, *ω*/*R*_*n*_, for *R*_*l*_/*R*_*n*_ = 0, 4, and 8. Since these curves all take *f* = 0.3 and *R*_*a*_ = 0, their current densities all approach 0.7 in the high-frequency limit given by Eq. (13).

Figure 1 illustrates the frequency-dependent conductivity of Eq. (13). The dashed curve shows that the conductivity rises monotonically with increasing frequency in the absence of carriers liberated from traps, *R*_*l*_/*R*_*n*_ = 0. By contrast, the solid curves depict the liberation of carriers from traps generating a substantial range of frequencies over which the conductivity falls with increasing frequency. As evidenced in this figure, the relatively mild frequency dependence of the conductivity observed experimentally occurs when *ω*/*R*_*n*_ ≫ 1.

A relatively simple expression for the slope of the relative conductivity *∂* [*σ*(*ω*)/*σ*_*n*_]/*∂*[*ω*/*R*_*n*_] is straightforwardly obtained from Eq. (13) in the relevant limits, *ω*/*R*_*n*_ → ∞, with vanishing transport through abnormal brain tissue, *R*_*a*_ → 0,

$$\frac{\partial \left[\sigma \left(\omega \right)/\sigma_{n}\right]}{\partial \left(\omega /R_{n}\right)}\to 2{\left(\frac{R_{n}}{\omega }\right)}^{3}\left[\left(1-f\right)-f\left(\frac{R_{l}}{R_{n}}+\frac{{R_{l}}^{2}}{{R_{n}}^{2}}\right)\right].$$ (17)

The sign of the slope is given by the sign of the square-bracketed term of Eq. (17). As illustrated by the dashed curve in Fig. 1, the slope is positive in the absence of ions being transferred from being trapped in abnormal tissue to being mobile in normal tissue, *R*_*l*_ = 0. By contrast, a negative slope is obtained when the second term within the square brackets of Eq. (17) dominates,

$$f>1/\left[1+\left(\frac{R_{l}}{R_{n}}\right)+{\left(\frac{R_{l}}{R_{n}}\right)}^{2}\right].$$ (18)

**FIG. 2.**
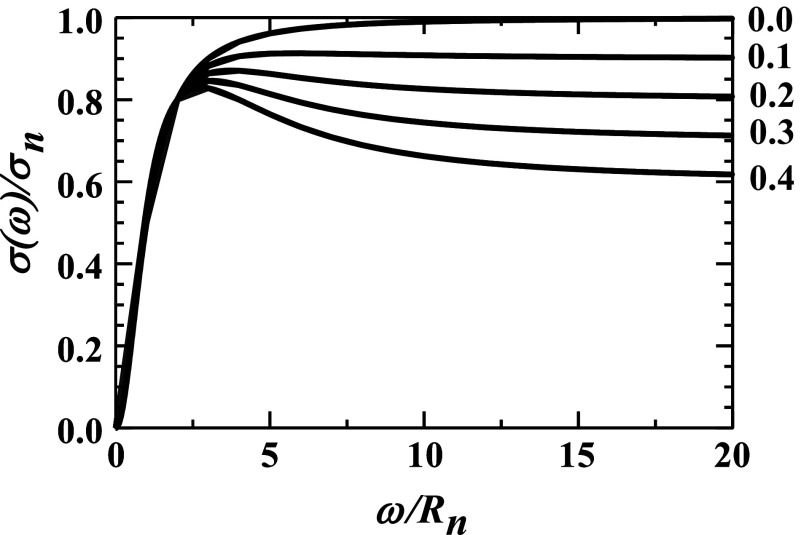
The relative conductivities, *σ*(ω)/*σ*_*n*_, from Eq. (13) are plotted vs the relative frequency, *ω*/*R*_*n*_, for increasing fractions of cations being trapped in abnormal tissue, *f* = 0.0, 0.1, 0.2, 0.3, and 0.4, with *R*_*l*_/*R*_*n*_ = 4 and *R*_*a*_ = 0. These curves all approach 1 − *f* in the high-frequency limit given by Eq. (13).

**FIG. 3.**
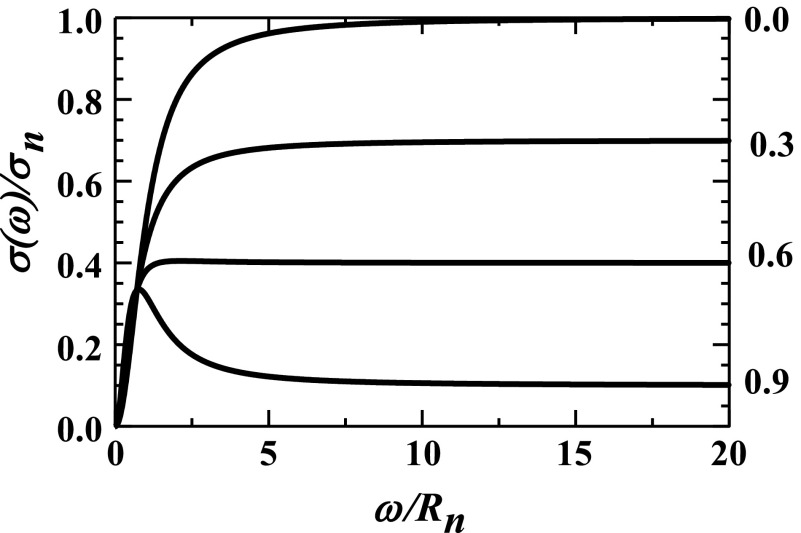
The relative conductivities, *σ*(ω)/*σ*_*n*_, from Eq. (13) are plotted vs the relative frequency, *ω*/*R*_*n*_, for increasing fractions of cations being trapped in abnormal tissue, *f* = 0.0, 0.3, 0.6, and 0.9 with *R*_*l*_/*R*_*n*_ = 0.5 and *R*_*a*_ = 0. These curves all approach 1 − *f* in the high-frequency limit given by Eq. (13).

Consider the two limiting situations that generate a negative slope. First, if *R*_*l*_/*R*_*n*_ ≫ 1, then, as illustrated in Fig. 2, a negative slope will result even when only a small fraction of ions is initially trapped in abnormal tissue, e.g., *f* ≪ 1. Second, if *R*_*l*_/*R*_*n*_ ≪ 1, then, as illustrated in Fig. 3, a negative slope only results when a large fraction of ions are initially trapped in abnormal tissue, *f* → 1 − (*R*_*l*_/*R*_*n*_). The high-frequency conductivity is then relatively small since it involves only a small fraction of tissue’s ions: *σ*(100 Hz) ≈ *σ*_*n*_(1 − *f*) ≈ *σ*_*n*_(*R*_*l*_/*R*_*n*_) ≪ *σ*_*n*_. Rather, most ions are initially trapped within abnormal tissue.

The second scenario appears most relevant to the observations reported in Refs. 4 and 5. In particular, (1) negative slopes are only observed in especially disorganized excised brain tissue and (2) these tissues’ high-frequency conductivities are lower than those of samples manifesting positive slopes.^5^

## III. SUMMARY AND DISCUSSION

Biological processes depend on the movement of ions. Ionic transport generally depends on the quality of the biological structures within which ions exist. The quality of these structures is evaluated by pathologists who relate it to the presence of disease. Charge transport studies can be combined with pathology investigations. For example, anomalous frequency dependences of the ionic conductivities are observed in brain tissues excised from pediatric epilepsy patients whose pathology examination reveals to be exceptionally disorganized.^5^

Here, we employ a simple model to describe changes in the ionic transport through brain tissue as a function of the extent of its structural abnormality. In particular, brain tissue is regarded as the mixture of normal tissue, which supports ionic transport, and abnormal tissue within which ions are trapped. As is widely observed, the ionic conductivity of typical freshly excised tissue *increases* slowly with the frequency of the applied electric field that drives cation transport. This behavior is understood as arising from ions moving until they encounter intrinsic blockages associated with cellular structures.^1^ By contrast, the conductivity of the unusually damaged material *decreases* with the frequency of the applied electric field.^5^ We attribute this anomalous behavior to electric-field induced freeing of cations from traps in abnormal tissue, thereby enabling the freed cations to move within normal tissue. In other words, the unusual frequency dependence of the conductivity is indicative of trap-limited ionic transport. As such, the densities of mobile ionic charge carriers are significantly smaller than the net density of these ions (mobile ions plus trapped ions).^5^

Solvated sodium cations are the predominant charge carriers in tissues excised from epilepsy patients.^1^ Excised tissues from the most severely affected patients tend to be most disorganized and to manifest conductivities that fall with increasing applied frequency to especially small (high-frequency) values.^5^ Nonetheless, the density of sodium nuclei measured by ^23^Na-MRI in epilepsy patients’ lesions is taken as indicative of the severity of this affliction.^6^ Taken together, the conductivity and ^23^Na-MRI measurements indicate that the density of trapped sodium cations rises with the severity of the structural disruptions associated with epilepsy. In other words, epilepsy’s etiology in these samples is consistent with (1) disorganized brain tissue, (2) high densities of trapped sodium atoms, and (3) low densities of mobile solvated sodium cations.

The qualitative features of these results are robust. However, we cannot estimate the fractions of excised brain tissue that should be regarded as normal and abnormal. Normal and abnormal tissues may be mixed within lesions. Furthermore, surgeons generally include some surrounding normal tissue when they excise a lesion. This effect will tend to exaggerate the fraction of normal tissue in an excised sample and its conductivity. As such, the prevalence of the distinctive features associated with abnormal tissue may be underestimated.

Our measurements do not allow us to reliably designate sodium cations as either intercellular or intracellular sodium cations. However, we note that the intercellular sodium concentration and volume fraction of the healthy rat brain (140 mM and 0.2) are quite different from the intracellular concentration and volume fraction (10 mM and 0.8).^8^

## Data Availability

The data that support the findings of this study are available within the article.
